# Shift to Virtual Self-Management Programs During COVID-19: Ensuring Access and Efficacy for Older Adults

**DOI:** 10.3389/fpubh.2021.663875

**Published:** 2021-05-31

**Authors:** Pamela Sanchez-Villagomez, Claudia Zurlini, Maggie Wimmer, Linda Roberts, Bertilia Trieu, Bonnie McGrath, Robyn Wiesel, Titilayo Ologhobo, Sandra Goldsmith, Laura Robbins

**Affiliations:** Education Institute, Hospital for Special Surgery (HSS), New York, NY, United States

**Keywords:** older adults, self-management, education, virtual, musculoskeletal health, exercise

## Abstract

**Background:** The COVID-19 pandemic resulted in significant uncertainty and disruption to many aspects of daily living, including physical activity, socialization opportunities, and access to healthcare services. Under these conditions, at-risk older adults are even more likely to be inactive and isolated, leading to potential exacerbation of musculoskeletal and chronic conditions and emotional distress. This case study provides an overview of our experience and best practices developed during our shift from onsite programming to virtual.

**Methodology:** HSS utilized varied online approaches, including phone/video conference classes, webinars, on-demand videos and email campaigns to successfully transition programs. Due to this shift, HSS changed its evaluation to an online approach, using a mixed method to adequately assess the impact of programs.

**Results:** Between April and August 2020, our virtual programs reached 428,766 participants, resulting in a 10,807% increase in program reach. Most participants assessed were 60 years or older (72%) and reported knowledge (85%) and self-management skills (83%) gained as well as high program satisfaction (90%). Analyses of program data did not show any statistical significant difference in self-reported health outcomes. However, qualitative results showed virtual programming helped to foster social connectivity during COVID-19, helped to build a daily routine, and positively impacted mental and physical health.

**Conclusion:** Shifting to virtual programming in the face of the pandemic enabled us to deliver effective programs affording our community the opportunity to stay physically active and socially connected despite the quarantine orders.

## Introduction

Musculoskeletal conditions are the most common cause of work-related disability among US adults (1). In the United States alone, 54.4 million adults have been diagnosed with arthritis (2). This problem increases with age—as nearly three out of four adults 65 years old and older are affected by musculoskeletal disease, the need to keep older adults active and informed is ever more present (3).

When COVID-19, the disease caused by the novel coronavirus (SARS-CoV-2), began escalating in New York City in March 2020, we knew there would be serious disruption to our community, as it includes a significant number of older adults. This was especially unfortunate because, although this population faces greater health risks due to COVID-19, they are also more affected by the negative consequences of a sedentary lifestyle (4).

Unable to leave home safely, cut off from friends, family, and support networks—the conditions surrounding the pandemic are an anathema to the health concerns of older adults, who require physical activity to maintain their mobility, independence, mental health, and well-being. In addition to a decline in musculoskeletal function, a sedentary lifestyle in older adults has been associated with high blood pressure, elevated cholesterol, cardiovascular disease, type II diabetes, and cancer as well as an increased risk of premature death (5–8). In comparison, Daskalopoulou and colleagues found that “higher levels of physical activity increase the odds of healthy aging by 39% (9).”

The physical changes and life transitions that present with age make older adults more vulnerable to social isolation (10), which has been identified with increased all-cause mortality as well as decreased cognitive function (11–13). This is a substantial public health issue, but one that can be at least partly addressed with the use of technology. However, many older adults face barriers to adapting to new technology (14). They may lack knowledge, confidence, or want additional guidance (15, 16). Others may be concerned about security and reliability (17). Yet research has shown these perceived limitations can be addressed with training (14–16). And, in fact, in a series of focus groups that included 113 older adults, Mitzner and colleagues found that positive attitudes about technology outnumbered negative attitudes (17).

The Public and Patient Education Department (PPED), part of the Education Institute at Hospital for Special Surgery (HSS), a large academic medical center specializing in musculoskeletal health, is committed to improving the health needs of culturally diverse communities, LGBTQ+ individuals, children, adults, and older adults who suffer from or are at risk of musculoskeletal and rheumatologic conditions. When the pandemic forced PPED to cancel onsite programming, we knew it would be essential to move access online and support our community through the transition, particularly those who relied on our institution's exercise and educational programming prior to COVID-19. This case study provides an overview of our experience and best practices developed during our initial shift from onsite programming to a virtual format, supporting and addressing the needs of older adults. This shift, reported herein, spanned from April to August 2020.

## Context

PPED runs a robust program of lectures\workshops, exercise classes, community outreach programs, support groups, and mind-body programs that have traditionally been attended primarily by residents of New York City and the Tri-state area. The focus of these initiatives is to help participants improve self-management of musculoskeletal health and thus improve quality of life.

Although virtual learning has become increasingly popular, in 2019, only 1% of our programming took place online. With over 3,900 community members participating in our programming, the majority (89.5%) were 60 years old or older (Table 1). Our engagement with older adults became even more urgent as the shut-down put more people at risk of negative health consequences from isolation and sedentary lifestyles—threats that are especially dangerous to the older adult population (6, 18). Our goal was to continue supporting our community by providing education and exercise to foster physical activity and socialization.

**Table 1:** Comparison of Participant Demographics in 2019 and 2020^a^.

	**2019 (%)**	**2020 (%)**
	***N* = 654**	***N* = 336**
**Gender**		
Female	78.4	88.0
Male	21.6	12.0
**Age (years)**		
Under 20	0.0	0.7
20–29	0.2	1.8
30–39	2.6	3.6
40–49	1.5	8.7
50–59	6.3	12.7
60–69	21.6	33.7
70–79	46.3	30.8
80–89	20.3	7.2
90+	1.3	0.7
**Race**^**b**^		
Black or African American	8.5	4.9
American Indian or Alaska Native	2.4	1.1
Asian	6.5	11.6
White/ Caucasian	77.1	81.3
Hawaiian or Pacific Islander	0.2	0.4
Other	8.0	1.9
**Ethnicity**		
Hispanic/Latino	46.1	7.0
Non-Hispanic/Latino	53.9	93.0
**Musculoskeletal Conditions**^**b**^		
Osteoarthritis	53.7	65.0
Rheumatoid Arthritis	14.7	10.9
Osteoporosis	49.4	39.9
Gout	3.5	2.2
Fibromyalgia	2.3	3.8
Other	23.6	27.9

*^a^Data reflects participants from April-August of each year.*

*^b^Does not sum to 100% due to multiple responses*.

With the shutdown, we had to decide which on-site programs we could move to a virtual setting and how best to do so. This transition needed to occur quickly, as many people had already enrolled in our programs. When that was accomplished, we were able to determine which additional needs we could address with programming during the pandemic. In response to the disruption to many aspects of daily living, along with access to health care services, we created short, on-demand videos. Finally, we pivoted to online evaluation of our programming, increasing our qualitative assessment efforts to ensure feedback from as many participants as possible.

### Programmatic Elements

During the first 5 months of the pandemic, we shifted 79% of our exercise and educational programming from on-site to virtual access. Safety and ability to deliver program content virtually were the primary reasons why we did not transition 100% of our scheduled programs to an online format.

#### Exercise Classes

Our exercise program consisted of five exercise classes (yoga, Yogalates™, Pilates, tai chi, and Dance for Fun and Fitness) that were led by certified fitness instructors specialized in working with older adults with musculoskeletal conditions. Each class is comprised of 12–15 participants and runs once a week for 60 min during 6-week increments. As we decided to move our exercise classes to a virtual format, we worked closely with our instructors, taking into consideration the popularity of the onsite class and, most importantly, safety of the exercise conducted without in-person instruction. We also notified participants of the shift by contacting them through email or phone.

Given our desire to provide continuity of services, there was a short turn-around time to start virtual exercise classes. So, we launched an aggressive training plan to ensure that program staff, instructors, and program participants were comfortable with using video conference platforms such as Skype and Zoom. For program staff, one staff member was identified and trained as the master user of Skype and Zoom. Afterward, the master user trained all other program staff and conducted various demonstrations to increase confidence among staff. For instructors, we provided one-on-one trainings and helped them arrange their teaching spaces to enable the best possible vantage point for their virtual studios. For our program participants, we created a four-page instruction guide with screenshots to help them access and navigate Skype, which was our initial platform for virtual classes. However, we found that this process added to participant confusion, with participants' personal computers or devices sometimes appearing different from the screenshots in our guide.

One of our greatest concerns was that older adults might struggle with the new technology, so we focused our attention to support them in this transition. Subsequently, as we transitioned from Skype to Zoom, we eliminated the guides and instead scheduled individualized Zoom “office hours,” for anyone who wanted assistance learning how to access the platform and/or to test the connection process. Two staff members were assigned as direct contacts for all class participants, and during off hours, we provided contact information to the Zoom help center. In addition, at the time of the initial transition, these same two program staff were also available during exercise sessions so that classes were not interrupted—one assisted instructors and facilitated the virtual streaming while the other was a direct contact for participants addressing their questions or issues. While there was a definite learning curve, our participants quickly transitioned to the new format and less staff were needed to support the programming.

#### Lectures/Workshops

Lectures are 60-min didactic sessions taught by physicians, nurses, physical, and occupational therapists and/or nutritionists. Workshops are 60–90 min sessions that offer interactive small group learning experiences covering topics such as good posture and approaches to pain and stress management. Pre-COVID, these programs were held in our New York City conference center or in a meeting space located in one of four Tri-state Outpatient Centers. In April, once the hospital acquired the necessary Zoom licenses, we transitioned onsite lectures to online webinars. The format changed from the academic style podium presentation style using PowerPoint slides that we used for onsite lectures to one that was more appealing for virtual audiences: a panel discussion led by a moderator. Programs were designed to keep participants engaged in the virtual environment and to provide them the same in-person opportunity of submitting their questions. These were live-streamed and recorded, resulting to 24 webinar recordings placed on YouTube for on-demand access. A staff member was also assigned to run the Zoom process and ensure a smooth experience. Given the interactive component of our workshops, we met with the facilitators to adapt the workshops so that they could be delivered safely and effectively in a virtual format.

#### Support Group

Traditionally, our support group for older adults met onsite once a month in New York City. When the pandemic hit, we increased its frequency to meet weekly but changed the format to conference calls. We also offered mind–body programming using the same method. These programs provided our community with much needed support during a time of isolation. Using conference calls as an alternative to implement programs was critical for us to meet the needs of our older adults as well as our underserved community that did not have a computer, tablet, or smart phone.

#### Informational Videos

Before COVID-19, we developed occasional videos focused on managing specific musculoskeletal conditions. These videos were heavily produced and took a while to complete. Since the onset of the pandemic, we decided to produce short videos (~5–6-min long) that could be accessed on demand at our YouTube channel. Over 5 months, we produced 11 of these videos, focused on topics that can help our extended community during a crisis. The only “production” these videos required was a short script from the instructor and a cell phone camera. Upon choosing a topic, we helped instructors develop a script, set their home “stage,” and practice delivery. Examples included: stress reduction, meditation for anxiety, and tips for the prevention of home exercise injuries. Ultimately, these videos provided bite-sized information for the consumer geared towards providing support in a variety of ways.

#### Community Outreach Programs

Before the pandemic, we brought education and exercise programs to diverse, underserved communities in New York City and CT, serving children, adults and older adults, many with limited English proficiency. This involved partnerships with local organizations and programming often took place at community sites, rather than at one of our facilities. Unfortunately, during the pandemic outreach was limited as many of the organizations we worked with were closed. We did consult with our community partners to determine feasibility of delivering programs *via* Zoom or conference call. Ultimately, in 2020 we were able to set up 13 virtual outreach programs, which was a significantly lower number than the 104 we provided in 2019 (Table 2).

**Table 2:** Comparison of number of programs and participants reached by program type in 2019 and 2020^a^.

**Program Type**	**2019 (*N*)**	**2020 (*N*)**
**Lectures/Workshops**		
Programs	32	54
Attendees	1,484	731
**Exercise classes**		
Programs	140	200
Attendees	830	2,214
**Support groups**		
Programs	22	41
Attendees	96	372
**Community outreach programs**		
Programs	104	13
Attendees	1,408	142
**Informational Videos**		
Videos	9	35
Viewers	113	425,307

*^a^Data reflects participants and programs from April-August of each year*.

#### Marketing

We expanded our marketing approach to reach a larger audience. Since we shifted our programs to a virtual format, we were able to accommodate a wider reaching community. Expansion included weekly email campaigns marketing our exercise and education programs and monthly paid Google ads marketing our YouTube videos. This is in addition to the traditional methods we used including print publications and social media. Beginning in late March, weekly email campaigns were sent to roughly 1,200 community members sharing health information relevant to the current situation, along the themes of mental health, exercising in quarantine, working from home, nutrition and more. Written in an easy-to-follow and engaging format, these emails also directed readers to additional resources such as our livestream and on-demand programming. Google ads were used to increase awareness of our growing YouTube playlist of short informational videos and webinar recordings.

#### Evaluation

Prior to the pandemic, we used a mixed-method approach—quantitative and qualitative strategies to evaluate the impact of our exercise classes. Greater emphasis was placed on our quantitative approach in distributing paper surveys in-person resulting in an average survey response rate of 60–70% per program. However, the change to an online program format necessitated a change to an online evaluation methodology, accomplished through email-administered surveys. But this led to a reduction in our average response rates, which fell to 20–30% per program. As a result of our limited quantitative data, it was critical to understand our community's needs, so we expanded our qualitative evaluation efforts to effectively assess the impact of our classes in its new format.

**Figure 1:**
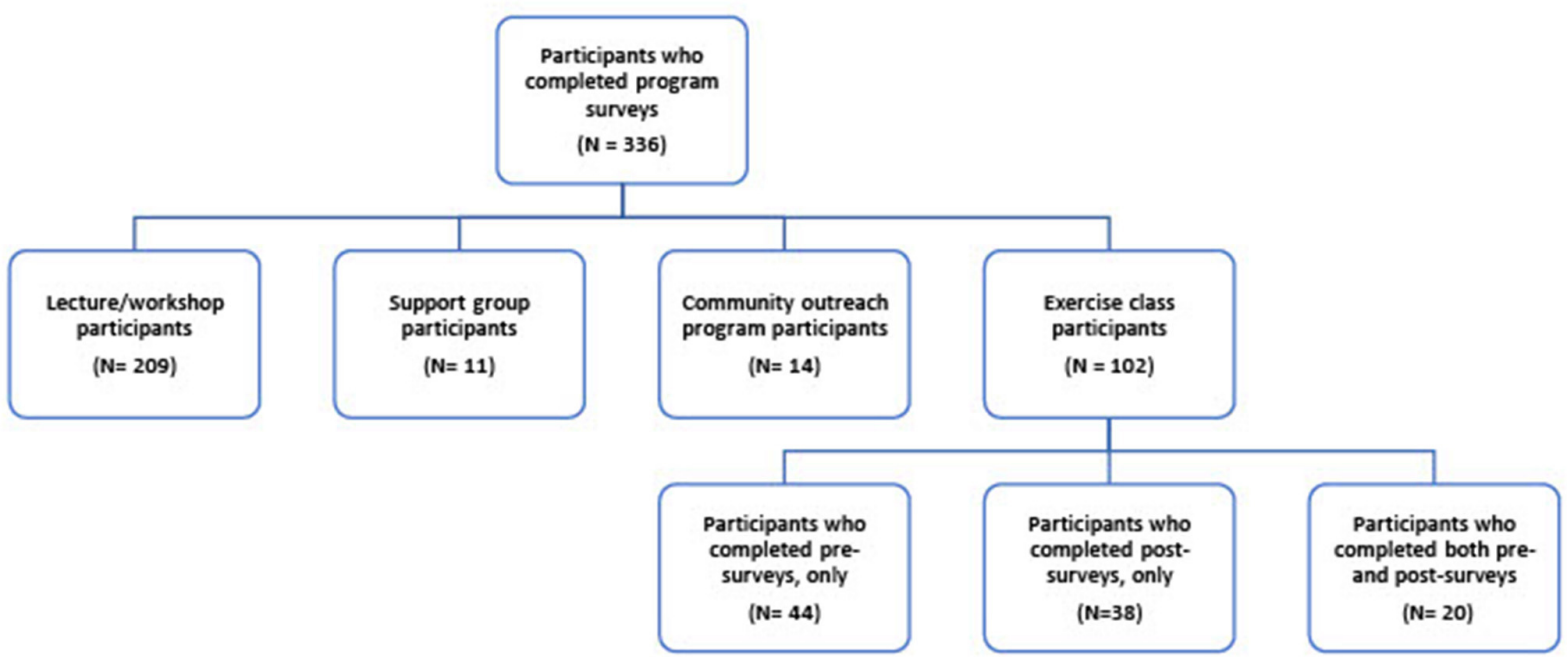
Quantitative survey response flowchart.

For quantitative analysis, we assessed demographic information, self-management skills, knowledge gain, program satisfaction, and change in self-reported health outcomes (such as pain intensity, pain interference, physical function, levels of stiffness and fatigue, self-efficacy to overcome barriers to physical activity and physical activity levels). Participants who signed up for 6-week exercise classes were asked to complete pre- and post-online surveys, while those who attended lectures/workshops, support groups, and community outreach programs were asked to only complete post-online surveys (Figure 1). Descriptive analyses, paired-sample *t*-tests and McNemar tests were conducted using SPSS 27 at 0.05 level of significance and 95% confidence interval.

**Figure 2:**
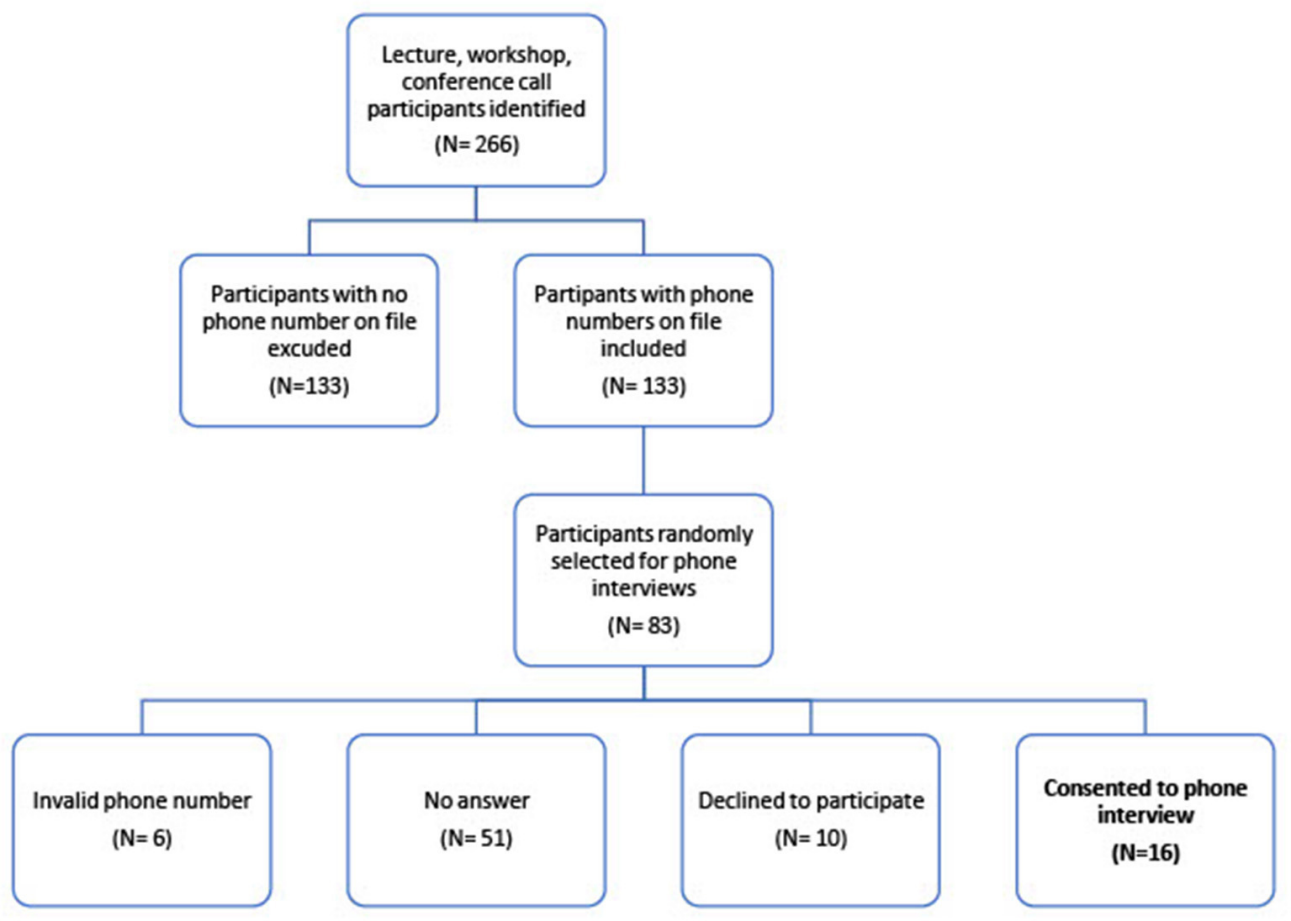
Participant selection for Qualitative Phone Interviews.

For qualitative analysis, we conducted phone interviews and focus groups, and implemented open-ended survey questions to assess participants' experience attending virtual programs, comfort with online learning platforms, willingness to attend in-person programs, and level of satisfaction with the program experience. Demographic information was not collected to protect participant privacy and confidentiality. From a sample of 133 program attendees, 83 were called (Figure 2). Of these, 57 did not answer the phone or had an incorrect number on file, 10 declined to participate and 16 consented to a phone interview. Reasons for which program attendees declined to participate were not recorded. We conducted eight focus groups, which had a total of 36 participants. In addition, 46 open-ended questionnaire responses were analyzed from the post-program surveys. Talking scripts were developed and used during semi-structured phone interviews and the focus groups. A specialized qualitative software (Dedoose 8.3.35, Hermosa Beach, California) was used to assign codes, develop categories, and evolving themes. A team of three HSS staff, external to the program, with expertise in qualitative research reviewed transcripts and conducted independent coding to develop validity and reliability of the data, as well as ensure integrity, consistency, and agreement between reviewers. The group discussed differences in code interpretations and developed a set of unifying themes.

IRB approval for human subjects research was obtained.

#### Impact of Virtual Programs

Between April and August 2020, we reached 428,766 participants through 343 virtual programs compared to 3,931 participants reached through 307 programs in 2019 of the same time period (Table 2). The majority of our participants were women (88%), 60 years and older (72%), and Caucasian (81%), and more than half self-reported that they had been diagnosed with at least one musculoskeletal health condition, i.e., osteoarthritis, osteoporosis, rheumatoid arthritis, gout, or fibromyalgia (Table 1). Of the exercise class participants (*N* = 20) who completed both pre- and post-online surveys, the majority were women (100%), 60 years or older (85%), non-Hispanic/Latino (95%), and Caucasian (94%).

Results from online post-surveys (*N* = 234), showed that lectures/workshops, support groups, and community outreach programs were also successful, as 85% of attendees reported that the program had increased their understanding of the topic, 83% reported that the information provided had added to their self-management skills and 90% “agreed” or “strongly agreed” when asked if they were satisfied with the program. Findings from exercise class pre-/post-online surveys (*N* = 20) did not show any significant differences in self-reported outcomes measures such as pain intensity, pain interference, physical function, fatigue and stiffness levels, self-efficacy to overcome barriers to physical activity, and physical activity levels (Table 3).

**Table 3:** Differences in Outcome Measures Between Pre and Post Intervention (*N* = 20)^a^.

**Measure**	**Pre-test**	**Post-test**	***P*-value^**b**^**
**Physical Function**^**c**^			
Ability to lift or carry groceries (*n*, %)	13 (65.0)	14 (70.0)	1.000
Ability to climb one flight of stairs (*n*, %)	15 (75.0)	17 (85.0)	0.500
Ability to climb several flights of stairs (*n*, %)	12 (63.2)	13 (68.4)	1.000
Ability to bend, kneel or stoop (*n*, %)	13 (65.0)	14 (70.0)	1.000
Ability to bath or dress yourself (*n*, %)	17 (89.5)	17 (89.5)	1.000
**Physical Activity Assessment**^**d**^			
Walking; ≥3 times/week for 30 min (*n*, %)	10 (50.0)	14 (70.0)	0.289
Moderate-intensity PA; ≥3 times/week for 30 min (*n*, %)	9 (47.4)	13 (68.4)	0.344
Vigorous-intensity PA; ≥3 times/week for 20 min (*n*, %)	0 (0.0)	7 (35.0)	-
Stiffness (M, SD)	2.2 (2.1)	1.7 (2.0)	0.268
Fatigue (M, SD)^e^	1.8 (2.2)	1.2 (1.6)	0.131
Self-Efficacy to Overcome Barriers to Physical Activity (M, SD)^f^	7.7 (2.4)	8.0 (2.4)	0.546
Pain Intensity (M, SD)^g^	2.3 (2.7)	1.8 (2.2)	0.523
**Pain Interference on aspects of quality of life**^**h**^			
General activity (M, SD)	0.9 (1.8)	0.8 (1.4)	0.691
Mood (M, SD)	0.6 (1.5)	0.8 (1.7)	0.330
Walking ability (M, SD)	1.1 (2.0)	0.8 (1.4)	0.349
Normal work (M, SD)	0.9 (1.8)	0.8 (1.4)	0.772
Relations with other people (M, SD)	0.6 (1.2)	0.5 (1.2)	0.494
Sleep (M, SD)	1.0 (1.9)	1.1 (2.1)	0.578
Enjoyment of life (M, SD)	0.7 (1.7)	0.7 (1.8)	1.000

*^a^Data are reported in mean (M), standard deviation (SD), number, and frequency.*

*^b^Statistical significance is based on McNemar and paired T-Tests statistics.*

*^c^As measured by the physical function items on the SF-36 Health Survey.*

*^d^As measured by the 3-question Physical Activity Questionnaire (3Q).*

*^e^As measured by the Exercise Regularly Scale of the Chronic Disease Self-Efficacy Scales.*

*^f^As measured by the Brief Fatigue Inventory.*

*^g^As measured by the PROMIS Numeric Rating Scale v1.0—Pain Intensity 1a.*

*^h^As measured by the Brief Pain Inventory*.

Results from qualitative analysis demonstrated appreciation of virtual programming by participants, as many considered it a wonderful experience and hoped it would continue even after onsite programming resumes. They welcomed the ability to include the addition of routinely scheduled online programs when many of their day-to-day activities were canceled due to COVID-19. They appreciated the opportunity for socialization with other class members and instructors and reported that the interpersonal connection improved their mental health. Although participants were uncomfortable about the safety of in-person classes once onsite programming resumes, many admitted that they had trust in HSS to maintain a safe environment, but not in the public transportation they would use to get there. They valued the convenience of the model and stated that with the assistance of our team, the platforms were easy to use. Participants noted that they would like more flexibility to interact and engage with instructors and classmates; they would also like to access more recordings of exercise classes (Table 4).

**Table 4:** Select Quotes from Participants in PPED Virtual Programming, April—August, 2020.

**Major Themes**	**Participant Feedback**
Establishing Routine	“The programs are providing a sense of normalcy and giving some structure to the day.”
	“The classes have given structure to my otherwise limited life under quarantine and provided a space for self-nurturing and self-care.”
	“It is a regular activity to look forward to while I'm stuck at home.”
Interpersonal Connection—Socialization	“The classes have given structure to my otherwise limited life under quarantine and provided a space for self-nurturing and self-care.”
	“It is a regular activity to look forward to while I'm stuck at home.”
Interpersonal Connection—Mental Health	“Helped to have an enjoyable time with others. Took me ‘out of my head' and helped me to regain more optimistic view for the future.”
Safety—Not comfortable with in-person	"In terms of going back to [in person] classes, I'm concerned about the cleanliness of the exercise equipment (like blocks and yoga mats, etc.). It's hard to know who else has used it and how well it has been cleaned.”
	“I have no desire to be back in a group environment, especially when there is a virtual option available. Why would I take a risk with my health?”
Safety—Trust in HSS	“I don't think it is a matter of holding in-person classes in HSS because I trust HSS to put safety measures in place. It is a matter of how I would get to the classes.”
Ease of Virtual Programming—Comfortable with technology	“I am not a computer person. I have a lot of problems with computers. However, HSS made it very easy for me and I had a very good experience.”
	“It was organized very well. [The coordinator] introduced [the instructor] and set us up. It took about a week or so to get everyone sorted out and then after that it went very smoothly. I could hear from the other participants and know they were happy too.”
Ease of Virtual Programming—Convenience of programs	“It has given me the opportunity to exercise when circumstances prevent me from going outdoors.”
	“It has helped me keep a regular schedule for exercising and has encouraged me to practice Tai Chi Chih on my own at home.”
More Dynamic Content Delivery—Increase interaction and engagement	“Online is not the same as in person….I miss [the instructor] telling us what we're doing incorrectly.”
	“When the support group is in-person we can have personal conversation after the group, but you can't do this when we are not in person.”
	“I think the program is good; but the class is only 1 h and could be extended for 5 min either before or after so you have a chance to ask questions. When you attend in person classes you have the opportunity to ask questions before or after the class.”
Appreciative of Programs—Positive Experience	“The fact that they exist is wonderful. I'm learning things that I haven't done before. I was poised to do Pilates before the pandemic so I found this to be an interesting way to extend into a new practice. It is an easy way to try something new or to do something that you've already been doing.”
Appreciative of Programs—Continuing Virtual Programs	“I hope it doesn't disappear because it is convenient—It has value and it will continue to have value when we go back to in-person.”
	“I would hope that when the city and state allow in-person classes the zoom classes be offered as something complementary to the in-person classes.”

## Discussion

Overall, results show that our programs remained popular with older adults, even when running on a virtual platform. Participants used our exercise classes to maintain physical activity, support mobility, relieve stress, and stay connected. Ultimately 90% of participants reported satisfaction with their experience. Recent studies have found virtual education can be effective in providing older adults with opportunities for exercise and improving pain management skills (19–21). Our research further supports these findings and shows that virtual programming can improve the quality of life of older adults.

The success of our online programming supports the value of this modality in the health education of older adults. *Older Americans 2020* reported that as people age, they spend less time socializing and being active. In fact, in 2018, only 14% of people age 65 and over met recommended guidelines on physical activity (22). Our research shows that with attention and flexibility, most older adults can become comfortable using online platforms. They can derive significant benefit from the ability to attend a workshop on posture or participate in a yoga class from the safety of their own homes. Furthermore, virtual programming enables older adults to access advice from the top experts on any topic from anywhere in the world.

In dealing with the demands of the situation, we learned to quickly adapt, prioritize, and move forward. We had been working on increasing our online presence, and the details of each step were meticulously planned and addressed. However, with the shutdown, our team needed to learn quickly and pivot when change was necessary. Due to the unique differences in our programs, we customized our approach to transitioning for each program by assessing the needs of our instructors/speakers and participants and identifying challenges and/or potential barriers. As we became more adept at delivering virtual programming, we worked toward extending the reach of our programs beyond our existing audience. Within 5 months, we achieved a 10,807% increase in program reach with participants from around the world accessing our virtual programs and our curated library of YouTube content. We plan to continue building on-demand content for YouTube after we return to in-person programming and continue offering many of our programs in a virtual format.

### Lessons Learned and Practical Implications

Throughout the process of switching to virtual formatting, we developed best practices and learned valuable lessons.

#### Exercise Classes

By offering classes online, we were able to expand our community far beyond the previous limits of New York City and its surrounding areas. Our most important consideration was to decide which classes could be offered virtually without risk to the participants (i.e., falls). However, it was also essential to work with all constituents and ensure that they are prepared and comfortable with the change in format through trainings. Ultimately, most participants found it easy and convenient. However, through evaluations, we learned that some participants wanted more time to socialize with other participants and instructors.

#### Lectures/Workshops

The first critical change was to revise the format of our onsite lectures to panel-driven interactive webinars to keep the audience engaged. Including a moderator helped to foster interactivity. We also developed topics that were grounded in the basics of musculoskeletal health but tailored for the particular moment. For example, one webinar discussed telehealth for musculoskeletal needs and another addressed ergonomics while working from home. We found it important to get the professional perspective of panelists from the beginning stages of program development. In planning, for example, they were best prepared to advise whether slides would be helpful or if a topic might be best addressed with a moderated discussion. We increased our reach by recording the programs and posting them on YouTube.

Finally, attendance was an issue. Before the COVID-19 shutdown, more than 50% of those who preregistered attended onsite lectures. While we saw higher registration numbers for our online programming, attendance rates dropped to roughly 30%. More research is needed to understand why attendance rates dropped. Overall, some people continued to have trouble with Zoom; some chose not to join by video and preferred to call into the Zoom number. Ultimately, we still had to offer some programs *via* conference call, specifically our support group for older adults and our mind–body workshops to manage pain and stress.

#### Community Outreach Programs

In navigating the circumstances with our community partners, we did our best to remain flexible—for example, being willing to use conference calls to deliver content. We also found it wise to plan for programs lasting a little longer than scheduled, as it often took some time to establish virtual or phone connection with community members.

#### Informational Videos

Videos were a significant part of our success in increasing viewership and reaching a large number of our community members; this was helped by paid advertising (i.e., Google ads). By the end of 2020, we achieved over 1.5 million viewers of our on-demand YouTube content. However, delivering appealing content was also key to our reach. One limitation was the inability to get demographic information from our YouTube viewers.

#### Evaluation

The move to online programming affected our survey response rates for exercise classes, with only 20 participants completing both pre- and post-online surveys. The small sample size was a limitation resulting in the inability to detect an effect and the magnitude of the effect. However, this limitation was addressed by enhancing our qualitative efforts to provide relevant and impactful data. There is a need to further explore effective online evaluation strategies to improve survey response rates and to engage in ongoing process improvement of program evaluation activities. Also, while we hoped to recruit a group with good racial and gender diversity, most of the participants were Caucasian women. We will continue to explore ways to improve diversity of participants.

## Conclusion

In the face of the pandemic, the ability to quickly move our programming to a virtual platform enabled PPED to deliver programs that enabled at-risk older adults to virtually participate in community education programs and exercise classes, despite quarantine orders. Our virtual programs aided in promoting musculoskeletal health, physical activity, and social connectedness, from the safety and comfort of participants' homes. Nonetheless, there is a need to further explore opportunities for additional socialization in online programming. Overall, this experience has shown that with careful planning, a shift in program delivery for older adults can be successful when accounting for perceived barriers to participation; and when programs are tailored to the specific needs (i.e., health, technology, access) of our community.

## Data Availability Statement

The original contributions presented in the study are included in the article/supplementary material, further inquiries can be directed to the corresponding author/s.

## Ethics Statement

The studies involving human participants were reviewed and approved by HSS Institutional Review Board. Written informed consent for participation was not required for this study in accordance with the national legislation and the institutional requirements.

## Author Contributions

PS-V, CZ, MW, LR, BT, BM, RW, TO, SG, and LR made substantial conceptual or design contributions or gathered and analyzed important data. PS-V, CZ, BT, TO, and SG either helped draft or critically revise the paper in keeping with important intellectual content. PS-V, CZ, MW, LR, BT, BM, RW, TO, SG, and LR provided final approval before publishing. PS-V, CZ, MW, LR, BT, BM, RW, TO, SG, and LR agreed to be accountable for the accuracy of the work. All authors contributed to the article and approved the submitted version.

## Conflict of Interest

The authors declare that the research was conducted in the absence of any commercial or financial relationships that could be construed as a potential conflict of interest.
